# Structural analysis of the full-length gene encoding a fibronectin-binding-like protein (CadF) and its adjacent genetic loci within *Campylobacter lari*

**DOI:** 10.1186/1471-2180-9-192

**Published:** 2009-09-08

**Authors:** Junichi Hirayama, Tsuyoshi Sekizuka, Akihiro Tazumi, Ikue Taneike, John E Moore, B Cherie Millar, Motoo Matsuda

**Affiliations:** 1 Laboratory of Molecular Biology, School of Environmental Health Sciences, Azabu University, Fuchinobe 1-17-71, Sagamihara 229-8501, Japan; 2 School of Biomedical Sciences, University of Ulster, Cromore Road, Coleraine, Co. Londonderry, N Ireland, BT52 1SA, UK; 3 Department of Bacteriology, Northern Ireland Public Health Laboratory, Belfast City Hospital, Belfast BT9 7AD, N. Ireland, UK

## Abstract

**Background:**

The combined sequences encoding a partial and putative *rpsI *open reading frame (ORF), non-coding (NC) region, a putative ORF for the *Campylobacter *adhesin to fibronectin-like protein (*cadF*), a putative Cla_0387 ORF, NC region and a partial and putative Cla_0388 ORF, were identified in 16 *Campylobacter lari *isolates, using two novel degenerate primer pairs. Probable consensus sequence at the -35 and -10 regions were identified in all *C. lari *isolates, as a promoter.

**Results:**

Thus, *cadF *(-like) gene is highly conserved among *C. lari *organisms. Transcription of the *cadF *(-like) gene in *C. lari *cells *in vivo *was also confirmed and the transcription initiation site was determined. A peptidoglycan-associating alpha-helical motif in the C-terminal regions of some bacterial cell-surface proteins was completely conserved amongst the putative *cadF *(-like) ORFs from the *C. lari *isolates.

**Conclusion:**

The putative *cadF *(-like) ORFs from all *C. lari *isolates were nine amino acid larger than those from *C. jejuni*, and showed amino acid residues 137 -140 of FALG (50% identity), instead of the FRLS residues of the maximal fibronectin-binding activity site demonstrated within *C. jejuni *CadF. A neighbor joining tree constructed based on *cadF *(-like) gene sequence information formed a major cluster consisting of *C. lari *isolates, separating from the other three thermophilic campylobacters.

## Background

Thermophilic *Campylobacter *species, primarily *Campylobacter jejuni *and *C. coli*, are curved, Gram-negative organisms, belonging to the ε-*Proteobacteria*, and are the most commonly recognized cause of acute bacterial diarrhea in the Western world [1-3].

*Campylobacter lari *is a relatively recently discovered thermophilic *Campylobacter *species that was first isolated from mammalian and avian species, particularly seagulls of the genus *Larus *[1,4]. *C. lari *has also been shown to be a cause of clinical infection [5-9].

In addition, an atypical group of isolates of urease-positive thermophilic *Campylobacter *(UPTC) have been isolated from the natural environment in England in 1985 [10]. Thereafter, these organisms were described as a biovar or variant of *C. lari *[11,12]. Subsequent reports described four human isolates in France [11,13]. Some additional isolates of UPTC have also been reported in Northern Ireland [14-16] in The Netherlands [17] and in Japan [18,19]. Thus, these two representative taxa, namely urease-negative (UN) *C. lari *and UPTC occur within the species of *C. lari *[20].

Bacterial pathogens have the ability to bind to fibronectin (Fn; a component of the extracellular matrix) [21-24]. Konkel *et al*. identified and cloned a gene encoding a fibronectin-binding protein (*Campylobacter *adhesin to Fn; CadF) from *C. jejuni *[22]. In *C. jejuni *and *C. coli*, the *cadF *virulence gene encodes a 37 kDa outer membrane protein that promotes the binding of these pathogens to intestinal epithelial cells [15].

In relation to *cadF *of thermophilic *Campylobacter *other than *C. jejuni *and *C. coli *described above, *cadF *and outer membrane protein gene F (*OprF*) have been identified in *C. coli *RM2228 (DDBJ/EMBL/GenBank accession number AAFL01000010 and ZP_00368187), *C. lari *RM2100 (AAFK01000002 and YP_002574995) and *C. upsaliensis *RM3195 (AAFJ01000008 and ZP_00371707), following whole genome shotgun sequence analysis [26]. However, no detailed descriptions of the *cadF *(*oprF*) gene have yet appeared for these thermophilic *Campylobacter *strains. In addition, no reports on the *cadF *(-like) gene in *C. lari *organisms have yet appeared.

Therefore, the aim of the present study was to clone, sequence and analyze the full-length gene encoding the Fn-binding (-like) protein (CadF) and its adjacent genetic loci from several *C. lari *organisms (UN *C. lari *and UPTC). We also aimed to confirm the expression of the gene in the *C. lari *cells.

## Results

### TA cloning, sequencing and sequence analyses of the full-length *cadF *gene and its adjacent genetic loci from the 16 isolates of *C. lari*

**Figure 1:**
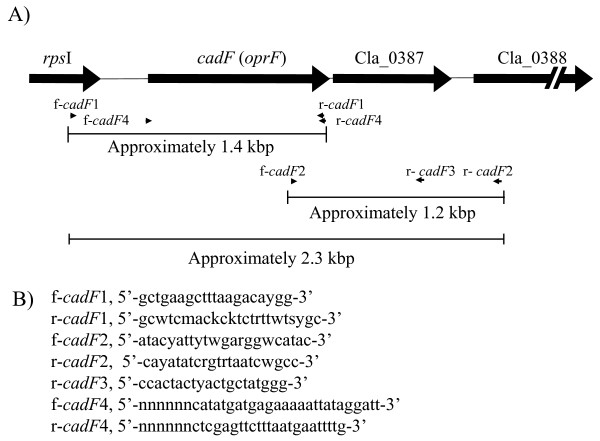
**A schematic representation of the *cadF *gene and its adjacent genetic loci for *C. lari *RM2100, including locations of the novel primers designed *in silico *(A)**. Nucleotide sequences of the primers are also shown (B).

The two primer pairs (f-/r-*cadF*1 and f-/r-*cadF*2; Figure1) successfully amplified PCR products of approximately 1.4 and 1.2 [kilo base pairs (kbp)], respectively, with all 16 isolates of *C. lari *employed (data not shown). Following TA cloning and sequencing, the combined nucleotide and deduced amino acid sequence data from the 16 isolates of *C. lari *determined have been made accessible in the DDBJ/EMBL/GenBank, with the accession numbers indicated in Table 1.

**Table 1:** *C. lari *isolates and other thermophilic *Campylobacter *reference strains analyzed in the present study and their accession numbers of the nucleotide sequence data accessible in DDBJ/EMBL/GenBank

**Isolate no**.	Source	Country	Accession number
*C. lari *JCM2530^T^	Seagull	Japan	AB465344
*C. lari *298	Human	Canada	AB465345
*C. lari *300	Seagull	USA	AB465346
*C. lari *84C-1	Human	N. Ireland	AB465347
UPTC 99	Sea water	N. Ireland	AB465348
UPTC NCTC12892	River water	England	AB295430
UPTC NCTC12893	River water	England	AB295431
UPTC NCTC12894	Sea water	England	AB295432
UPTC NCTC12895	Mussel	England	AB295433
UPTC NCTC12896	Mussel	England	AB295434
UPTC CF89-12	River water	Japan	AB295435
UPTC A1	Seagull	N. Ireland	AB295436
UPTC A2	Seagull	N. Ireland	AB295437
UPTC A3	Seagull	N. Ireland	AB295438
UPTC 89049	Human	France	AB295439
UPTC 92251	Human	France	AB295440
*C. lari *RM2100	Human	USA	AAFK01000002
*C. jejuni *NCTC11168	Human	USA	NC_002163
*C. jejuni *RM1221	Chicken	USA	NC_003912
*C. jejuni *81-176	Human	USA	NC_008787
*C. jejuni *260.94	Human	South Africa	AANK01000004
*C. jejuni *CF93-6	Human	Japan	AAFJ01000005
*C. jejuni *HB93-13	Human	China	AANQ01000001
*C. jejuni *84-25	Human	Unknown	AANT02000001
*C. jejuni *ss doylei 269.97	Human	Unknown	AARB01000000
*C. coli *RM2228	Chicken	USA	AAFL01000008
*C. upsaliensis *RM3195	Human	USA	AAFJ01000005

The combined sequences of an approximately 2.3 kbp region encoding a partial and putative ribosomal protein SI *rpsI *open reading frame (ORF) (165 bp), a NC region downstream of the ORF (approximately 250 bp), a putative *cadF *(-like) ORF (984 bp), a Cla_0387 ORF (642 bp), a NC region (approximately 120 bp) and a partial and putative Cla_0388 ORF (126 or 128 bp) were identified with all 16 *C. lari *isolates examined.

The present sequence analyses identified the putative ORF for *cadF *(-like) gene to be 984 bp [nucleotide position (np) 414-1,397 bp for the *C. lari *JCM2530^T^] with all 16 *C. lari *isolates (n = 4 UN *C. lari*; n = 12 UPTC) and UN *C. lari *RM 2100. With regard to the *cadF*-like gene, the sequence commenced with an ATG start codon for all isolates and terminated with a TAA for 13 isolates and with a TGA for the other three isolates (NCTC12894, 12895 and 99). Regarding putative ORFs for *cadF *(-like) gene, apparent size differences occurred amongst the four thermophilic *Campylobacter *species examined, 984 bp (328 amino acid residues) for 16 *C. lari *isolates and *C. lari *RM2100 strain, 957 (319) for *C. jejuni *RM1221 and NCTC11168, 996 (332) for *C. coli *RM2228, and 948 (316) for *C. upsaliensis *RM3195, as shown in Table 2, although in this limited study a small number of reference strains of *C. jejuni*, *C. coli *and *C. upsaliensis *were examined. Probable ribosome-binding (RB) sites, AGGA (np 404-407 bp) [Shine-Dalgarno (SD) sequences] [27], that are complementary to a highly conserved sequence of CCUCCU, close to the 3' end of 16S rRNA, were also identified in all the *C. lari *isolates examined.

**Table 2:** Putative ORFs of cadF (-like)and Cla_0387 genes from C. lariisolates and

	*cadF *(-like)			Cla_0387		
		
*Campylobacter*	ORF	Number of amino acid	CMW(Da)	ORF	Number of amino acid	CMW(Da)
*C. lari *JCM2530^T^	984	328	36,781	642	214	23,689
*C. lari *298	984	328	36,693	642	214	23,717
*C. lari *300	984	328	36,708	642	214	23,730
*C.lari *84C-1	984	328	36,578	642	214	23,689
UPTC 99	984	328	36,707	642	214	23,717
UPTC NCTC12892	984	328	36,674	642	214	23,695
UPTC NCTC12893	984	328	36,672	642	214	23,695
UPTC NCTC12894	984	328	36,695	642	214	23,695
UPTC NCTC12895	984	328	36,718	642	214	23,695
UPTC NCTC12896	984	328	36,836	642	214	23,845
UPTC CF89-12	984	328	36,817	642	214	23,692
UPTC A1	984	328	36,869	642	214	23,838
UPTC A2	984	328	36,869	642	214	23,838
UPTC A3	984	328	36,802	642	214	23,815
UPTC 89049	984	328	36,803	642	214	23,845
UPTC 92251	984	328	36,850	642	214	23,875
*C. lari *RM2100	984	328	36,707	642	214	23,689
*C. jejuni *NCTC11168	957	319	35,996	639	213	23,637
*C. jejuni *RM1221	957	319	35,998	639	213	23,794
*C. coli *RM2228	996	332	37,447	636	212	23,878
*C. upsaliensis *RM3195	948	316	35,624	648	216	24,279

ORF, open reading frame; CMW, calculated molecular weights; Da, daltons.

In the region upstream of the *cadF*-like gene, a most probable promoter consensus sequence at the -10 region (TATAAT) (TAGAAT for UPTC isolates (271-276 for UPTC CF89-12)) was identified at the locus between np 272 and 277 bp, with all 16 *C. lari *isolates and the *C. lari *RM2100 strain. In addition, probable -35 regions (np 243-248) upstream of the -10 region were also identified, in all *C. lari *isolates examined.

A putative ORF for the Cla_0387 gene was also estimated to be 642 bp with all 16 *C. lari *isolates examined (np 1,404 - 2,045 bp). The Cla_0387 gene commenced with a TTG and terminated with a TAA with all 16 *C. lari *isolates and the *C. lari *RM2100 strain. Apparent small size differences of the putative ORFs for the Cla_0387 also occurred amongst the four thermophilic *Campylobacter *species examined (Table 2).

As shown in Table 3, the nucleotide sequences of the full-length *cadF *(-like) structural gene from the 17 *C. lari *isolates showed 89.4-100.0% similarities to each other (Table 3). The nucleotide sequences of the full-length Cla_0387 structural gene from the 17 *C. lari *isolates showed 85.1 - 100.0% similarities to each other (Table 4). Thus, the nucleotide sequence similarities of the *cadF*-like gene appear to be slightly higher than those of the Cla_0387 gene, amongst the 16 *C. lari *isolates and the *C. lari *RM2100 strain examined.

**Table 3:** Nucleotide (upper right) and deduced amino acid (lower left) sequence similarities (%) of full-length *cadF *(-like) gene in *C. lari *isolates and other thermo- philic *Campylobacter *reference strains

	*Campylobacter*	1	2	3	4	5	6	7	8	9	10	11	12	13	14	15	16	17	18	19	20	21
1	*C. lari *JCM2530^T^		99.2	99.2	99.3	92.8	92.4	92.3	92.6	91.9	90.1	91.0	89.4	89.4	89.5	89.9	89.5	99.1	68.7	68.5	65.8	65.0
2	*C. lari *298	98.8		99.8	99.9	92.9	92.5	92.4	92.7	92.0	90.0	91.2	89.4	89.4	89.5	89.9	89.5	99.8	68.8	68.6	65.8	65.4
3	*C. lari *300	99.1	99.7		99.9	92.9	92.5	92.4	92.7	92.0	90.0	91.2	89.4	89.4	89.5	89.9	89.5	100.0	68.8	68.6	65.8	65.6
4	*C. lari *84C-1	99.1	99.7	100.0		93.0	92.6	92.5	92.8	92.1	90.1	91.3	89.5	89.5	89.6	90.0	89.6	99.9	68.9	68.7	65.9	65.5
5	UPTC 99	93.0	93.0	93.3	93.3		98.6	98.6	99.6	99.0	92.4	94.5	91.0	91.0	91.0	91.1	90.9	92.9	69.2	69.0	66.2	65.3
6	UPTC NCTC12892	93.0	93.0	93.3	93.3	99.1		99.4	98.1	97.5	92.1	94.0	90.9	90.9	90.9	91.0	90.8	92.5	68.9	68.7	65.7	65.4
7	UPTC NCTC12893	92.7	92.7	93.0	93.0	98.8	99.1		98.1	97.7	92.1	94.1	90.9	90.9	90.9	91.0	90.8	92.4	69.1	68.9	65.9	65.3
8	UPTC NCTC12894	92.4	92.4	92.7	92.7	99.4	98.5	98.2		98.6	92.1	94.4	90.7	90.7	90.7	90.8	90.6	92.6	69.1	68.8	66.1	65.3
9	UPTC NCTC12895	91.8	91.8	92.1	92.1	98.8	97.9	98.2	98.2		91.4	93.6	90.0	90.0	90.4	90.3	89.9	91.9	68.9	68.7	65.9	64.9
10	UPTC NCTC12896	90.9	90.9	91.2	91.2	95.4	94.8	94.5	95.4	94.2		91.9	98.0	98.0	98.4	98.3	98.5	90.1	68.3	68.2	66.3	65.0
11	UPTC CF89-12	91.8	91.8	92.1	92.1	95.4	94.8	94.5	95.4	94.5	93.3		91.3	91.3	91.2	91.4	91.2	91.2	69.2	69.1	66.3	65.6
12	UPTC A1	91.2	91.2	91.5	91.5	94.5	94.2	93.9	94.5	93.3	97.9	93.6		100.0	99.0	99.3	99.3	89.5	68.5	68.4	66.0	64.8
13	UPTC A2	91.2	91.2	91.5	91.5	94.5	94.2	93.9	94.5	93.3	97.9	93.6	100.0		99.0	99.3	99.3	89.5	68.5	68.4	65.8	64.8
14	UPTC A3	91.5	91.5	91.8	91.8	94.8	94.5	94.2	94.8	93.6	98.8	93.9	99.1	99.1		99.5	99.5	89.6	68.3	68.2	66.4	65.0
15	UPTC 89049	91.8	91.8	92.1	92.1	95.1	94.8	94.5	95.1	93.9	98.5	94.2	99.4	99.4	99.7		99.4	90.0	68.5	68.4	66.4	64.7
16	UPTC 92251	91.5	91.5	91.8	91.8	94.5	94.2	93.9	94.5	93.2	98.5	93.9	98.8	98.8	99.7	99.4		89.6	68.3	68.2	66.2	64.7
17	*C. lari *RM2100	99.1	99.7	100.0	100.0	93.0	93.3	93.0	92.7	92.1	91.5	91.8	91.2	91.2	91.5	91.8	91.5		68.9	68.7	65.9	65.6
18	*C. jejuni *NCTC11168	57.0	57.3	57.6	57.6	57.6	57.6	57.3	57.9	57.3	56.7	57.7	57.1	57.1	56.8	56.8	56.5	57.1		99.8	82.8	74.7
19	*C. jejuni *RM1221	56.4	56.7	57.0	57.0	57.3	57.0	56.7	57.6	57.0	56.7	57.4	57.1	57.1	56.8	56.8	56.5	56.5	99.4		82.6	74.5
20	*C. coli *RM2228	55.5	55.5	55.8	55.8	55.2	55.8	55.5	55.2	54.9	55.3	55.3	55.5	55.5	55.8	55.8	55.5	55.3	81.3	81.0		71.0
21	*C. upsaliensis *RM3195	53.6	54.0	54.2	54.2	54.0	53.6	53.6	54.2	53.6	53.6	54.9	53.5	53.5	53.8	53.8	53.8	54.1	74.1	73.8	68.6	

Moreover, the deduced amino acid sequence alignment analyses were also performed for putative ORFs of the full-length *cadF *(-like) gene of 16 *C. lari *isolates, as well as those of *C. lari *RM2100, *C. jejuni*, *C. coli *and *C. upsaliensis *strains. The putative ORFs from the 17 *C. lari *isolates showed 90.9 - 100.0% amino acid sequence similarities to each other, and 56.4 - 57.9% similarities, with those of two *C. jejuni *strains (Table 3). They also showed 53.5 - 55.8% similarities with those of other thermophilic *Campylobacter *organisms (two strains of *C. coli *and *C. upsaliensis*; Table 3).

Thus, the putative ORFs of the full-length *cadF *(-like) gene from the 17 *C. lari *isolates identified in the present study are identical size (984 bp and 328 amino acid residues) with sequence heterogeneity, at both nucleotide and amino acid levels.

As shown in Table 4, the deduced amino acid sequence similarities were also examined for the putative ORFs of the full-length Cla_0387 gene among the 17 *C. lari *isolates (86.9 - 100.0%) and other thermophilic *Campylobacter *organisms (50.7 - 56.2%), employed as references (data not shown).

**Table 4:** Nucleotide (upper right) and deduced amino acid (lower left) sequence similarities (%) of full-length CLA0749 in *C. lari *isolates and other thermophilic *Campylobacter*reference strains employed

	*Campylobacter*	1	2	3	4	5	6	7	8	9	10	11	12	13	14	15	16	17	18	19	20	21
1	*C. lari *JCM2530^T^		99.9	100.0	99.7	89.4	90.0	90.0	89.4	89.4	85.5	90.0	85.5	85.5	85.4	85.5	85.5	100.0	61.7	61.6	61.8	62.5
2	*C. lari *298	99.5		99.8	99.8	89.3	89.9	89.9	89.3	89.3	85.4	89.9	85.4	85.4	85.2	85.4	85.4	99.8	61.6	61.4	61.6	62.3
3	*C. lari *300	100.0	99.5		99.7	89.4	90.0	90.0	89.4	89.4	85.5	90.0	85.5	85.5	85.4	85.5	85.5	100.0	61.7	61.6	61.8	62.5
4	*C. lari *84C-1	99.5	100.0	99.5		89.1	89.7	89.7	89.1	89.4	85.2	89.7	85.2	85.2	85.1	85.2	85.2	99.7	62.2	62.1	61.6	62.3
5	UPTC 99	92.1	92.1	92.1	92.1		98.0	98.0	98.4	98.9	88.6	95.3	88.6	88.6	88.5	88.6	88.6	89.4	62.4	62.2	63.3	64.1
6	UPTC NCTC12892	93.0	93.0	93.0	93.0	99.1		100.0	97.7	97.8	89.4	95.1	89.1	89.1	89.2	89.4	89.4	90.0	61.8	61.6	63.1	64.1
7	UPTC NCTC12893	92.6	92.6	92.6	92.6	98.6	99.6		97.7	97.8	89.4	95.1	89.1	89.1	89.2	89.4	89.4	90.0	61.8	61.6	63.1	64.1
8	UPTC NCTC12894	92.5	92.5	92.5	92.5	98.1	99.1	98.6		98.9	88.2	95.0	88.2	88.2	88.0	88.2	88.2	89.4	61.6	61.4	62.8	63.4
9	UPTC NCTC12895	93.0	93.0	93.0	93.0	99.1	100.0	99.6	99.1		88.3	95.5	88.3	88.3	88.2	88.3	88.3	89.4	62.1	61.9	63.0	63.5
10	UPTC NCTC12896	87.4	87.4	87.4	87.4	90.2	90.2	89.8	89.7	90.2		87.7	99.1	99.1	99.8	100.0	99.8	85.5	63.4	62.9	63.2	64.4
11	UPTC CF89-12	92.5	92.5	92.5	92.5	96.7	97.7	97.2	97.2	97.7	88.8		87.7	87.7	87.5	87.7	87.7	90.0	63.0	63.7	63.8	64.0
12	UPTC A1	87.9	87.9	87.9	87.9	90.7	90.7	90.2	90.2	90.7	98.6	89.3		100.0	98.9	99.1	98.9	85.5	63.5	63.1	63.2	64.6
13	UPTC A2	87.9	87.9	87.9	87.9	90.7	90.7	90.2	90.2	90.7	98.6	89.3	100.0		98.9	99.1	98.9	85.5	63.5	63.1	63.2	64.6
14	UPTC A3	86.9	86.9	86.9	86.9	89.7	89.7	89.3	89.2	89.7	99.5	88.3	98.1	98.1		99.8	99.7	85.4	63.2	62.8	63.0	64.3
15	UPTC 89049	87.4	87.4	87.4	87.4	90.2	90.2	89.8	89.7	90.2	100.0	88.8	98.6	98.6	99.5		99.8	85.5	63.4	62.9	63.2	64.4
16	UPTC 92251	87.4	87.4	87.4	87.4	90.2	90.2	89.8	89.7	90.2	99.5	88.8	98.1	98.1	99.1	99.5		85.5	63.2	62.8	63.4	64.3
17	*C. lari *RM2100	100.0	99.5	100.0	99.5	92.1	93.0	92.6	92.5	93.0	87.4	92.5	87.9	87.9	86.9	87.4	87.4		61.7	61.6	61.8	62.5
18	*C. jejuni *NCTC11168	51.2	51.2	51.2	51.2	52.1	52.1	51.9	51.6	52.1	52.1	52.1	52.1	52.1	51.6	52.1	52.1	51.2		99.1	81.6	63.3
19	*C. jejuni *RM1221	50.7	50.7	50.7	50.7	51.6	51.6	51.4	51.2	51.6	51.6	51.6	51.6	51.6	51.2	51.6	51.6	50.7	98.6		81.4	63.6
20	*C. coli *RM2228	52.6	52.6	52.6	52.6	54.0	54.0	53.7	53.5	54.0	52.6	54.0	52.1	52.1	52.1	52.6	52.6	52.6	81.2	80.8		63.8
21	*C. upsaliensis *RM3195	54.2	54.2	54.2	54.2	55.6	55.6	55.6	56.2	55.6	55.3	55.1	55.3	55.3	54.0	55.3	55.3	54.2	55.6	55.6	56.9	

Thus, *cadF *(-like) gene is highly conserved among *C. lari *organisms isolated from humans and natural environments in several countries of Asia, Europe and North America.

In relation to the NC regions, two NC regions of approximately 250 bp, including a promoter at the -10 region and 120 bp occurred upstream of the *cadF *(-like) gene and downstream of the Cla_0387 gene, respectively, when examined combined sequences from all 16 *C. lari *isolates. Nucleotide sequences of approximately 250 bp from the 16 *C. lari *isolates and *C. lari *RM2100 showed 85.0 - 100.0% sequences similarities to each other (Table 5). Nucleotide sequences of approximately 120 bp also showed 85.6 - 100.0% sequence similarities among the 17 *C. lari *isolates. Thus, a considerable genetic heterogeneity of nucleotide sequences in the 250 bp NC region, full-length *cadF *(-like) gene, full-length Cla_0387 gene and the 120 bp NC region identified in the present study also occurred among the 17 *C. lari *isolates including the *C. lari *RM2100 strain.

**Table 5:** Nucleotide sequence similarities (%) of the NC regions upstream of *cadF (-*like) gene(250 bp; upper right) and downstream of Cla_0387 (120 bp; lower left) among *C. lari *isolates

	*Campylobacter lari*	1	2	3	4	5	6	7	8	9	10	11	12	13	14	15	16	17
1	*C.lari *JCM2530^T^		98.8	98.8	98.4	87.3	89.7	89.7	88.1	88.6	89.1	86.5	87.5	87.5	87.9	87.8	87.9	98.8
2	*C.lari *298	100.0		100.0	99.6	88.1	89.7	89.7	88.2	88.6	88.8	86.9	87.2	87.2	87.5	87.5	87.5	100.0
3	*C.lari *300	100.0	100.0		99.6	88.1	89.7	89.7	88.2	88.6	88.8	86.9	87.2	87.2	87.5	87.5	87.5	100.0
4	*C.lari *84C-1	100.0	100.0	100.0		87.8	89.3	89.3	87.8	88.2	88.4	86.5	86.8	86.8	87.1	87.0	87.1	99.6
5	UPTC 99	93.2	93.2	93.2	93.2		95.6	95.6	96.0	96.0	90.0	89.0	85.0	85.0	85.9	85.4	85.3	88.1
6	UPTC NCTC12892	93.2	93.2	93.2	93.2	98.3		100.0	96.8	97.6	91.3	89.7	86.6	86.6	87.0	87.0	87.3	89.7
7	UPTC NCTC12893	93.2	93.2	93.2	93.2	98.3	100.0		96.8	97.6	91.3	89.7	86.6	86.6	87.0	87.0	87.3	89.7
8	UPTC NCTC12894	93.2	93.2	93.2	93.2	100.0	98.3	98.3		98.4	93.2	89.0	86.3	86.3	86.7	86.6	87.0	88.2
9	UPTC NCTC12895	93.2	93.2	93.2	93.2	99.2	97.4	97.4	99.2		92.5	89.4	85.6	85.6	85.9	85.9	86.2	88.6
10	UPTC NCTC12896	88.1	88.1	88.1	88.1	92.4	90.7	90.7	92.4	91.5		86.5	92.3	92.3	92.7	92.7	93.1	88.8
11	UPTC CF89-12	89.7	89.7	89.7	89.7	91.5	91.5	91.5	91.5	90.6	85.6		85.5	85.5	85.5	85.4	85.7	86.9
12	UPTC A1	88.1	88.1	88.1	88.1	92.4	90.7	90.7	92.4	91.5	100.0	85.6		100.0	99.2	98.8	99.2	87.2
13	UPTC A2	88.1	88.1	88.1	88.1	92.4	90.7	90.7	92.4	91.5	100.0	85.6	100.0		99.2	98.8	99.2	87.2
14	UPTC A3	88.1	88.1	88.1	88.1	92.4	90.7	90.7	92.4	91.5	100.0	85.6	100.0	100.0		99.2	99.2	87.5
15	UPTC 89049	88.1	88.1	88.1	88.1	92.4	90.7	90.7	92.4	91.5	100.0	85.6	100.0	100.0	100.0		98.8	87.5
16	UPTC 92251	88.1	88.1	88.1	88.1	92.4	90.7	90.7	92.4	91.5	100.0	85.6	100.0	100.0	100.0	100.0		87.5
17	*C. lari *RM2100	100.0	100.0	100.0	100.0	93.2	93.2	93.2	93.2	93.2	88.1	89.7	88.1	88.1	88.1	88.1	88.1	

NC, non-coding.

### Northern blot hybridization, reverse transcription-PCR and primer extension analysis

**Figure 2:**
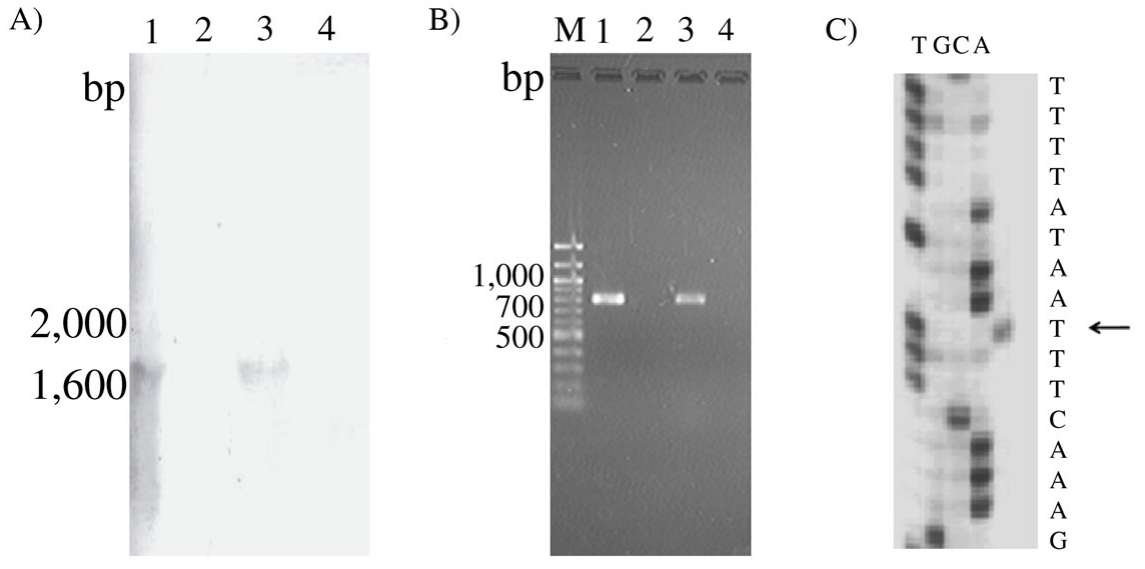
**Northern blot hybridization (A) and RT-PCR (B) analyses of the *cadF *(-like) and Cla_0387 structural gene transcripts expressed in the *C. lari *isolates**. Lane M, 100 bp DNA ladder; Lane 1, *C. lari *JCM2530^T ^with the reverse transcriptase (RTase); lane 2, *C. lari *JCM2530^T ^without the RTase.; lane 3, UPTC CF89-12 with the RTase; lane 4, UPTC CF89-12 without the RTase. Primer extension analysis (C) of the *cadF *(-like) and Cla_0387 mRNA transcript in the *C. lari *JCM2530^T ^isolate cells. The arrow indicates the transcription initiation site.

Northern blot hybridization analysis detected the *cadF *(-like) gene transcription in the two *C. lari *isolates cells, UN *C. lari *JCM2530^T ^and UPTC CF89-12 (Figure 2A). Since the positive signals of the hybridization were shown at around 1,600 bp (Figure 2A), the *cadF *(-like) gene may possibly be transcribed together with the Cla_0387 gene. Thus, *cadF *(-like) gene transcription was confirmed in the *C. lari *organisms. When RT-PCR analysis was carried out for the RNA components extracted from the UN *C. lari *JCM2530^T ^and UPTC isolates CF89-12 cells with the primer pair of f-*cadF*2 in the *cadF *(-like) gene and r-*cadF*3 in the Cla_0387 gene, as shown in Figure 1, a positive RT-PCR signal was detected at around 800 bp region with both isolates, respectively (Figure 2B).

The transcription initiation site for the *cadF *(-like) gene was determined by the primer extension analysis (Figure 2C). The +1 transcription initiation site for the *cadF *(-like) gene is underlined in the following sequence; 5'-TTTTATAATTTCAAAG-3', as shown in Figure 2C.

### Deduced amino acid sequence alignment analysis and phylogenetic analyses of the *cadF *(-like) ORF

**Figure 3:**
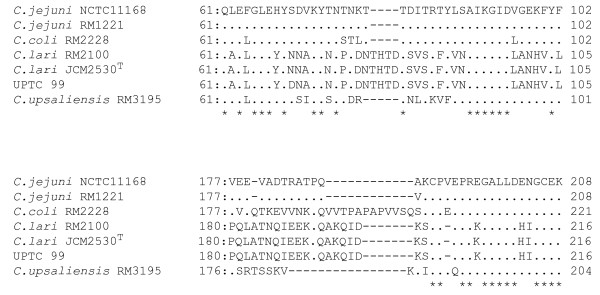
**Amino acid sequence alignment analysis of parts (around larger CadF sequences for *C. coli *and *C. lari*) of the putative *cadF *(-like) ORF from the thermophilic *Campylobacter *isolates examined in the present study**. Since nine amino acid larger sequences of the other 14 *C. lari *isolates were identical to either those from the *C. lari *JCM2530^T ^or UPTC isolates, alignment analysis data were omitted from the Figure.

We carried out deduced amino acid sequence alignment analysis to elucidate the differences in CadF (-like) protein amongst the thermophilic *Campylobacter*. As shown in Figure 3, the *C. coli *RM2228 strain carried a strech of 12 amino acid (VVTPAPAPVVSQ) from amino acid positions 190 to 201, as well as a Q at amino acid position 180, and regarding the nine larger amino acid for *C. lari *isolates than *C. jejuni *strains, four amino acid sequences (THTD) from amino acid positions 80 to 83 and five [A(T for UPTC 99) KQID] from 193 to 197 were identified to occur.

**Figure 4:**
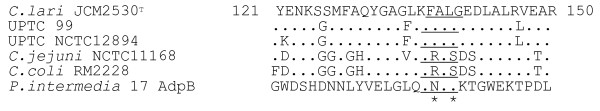
**Amino acid sequence alignment analysis of part (around a single-Fn binding domain within *C. jejuni *CadF) of the putative ORF for *cadF *(-like) gene from the 17 *C. lari *isolates**. Amino acid sequences of those from the *C. jejuni *and *C. coli *reference strains were aligned for comparison. FALG residues of *C. lari *and FRLS residues of *C. jejuni *and *C. coli *strains were underlined, respectively. In this Figure, amino acid sequence of AdpB (aa 201-230) from *Prevotella intermedia *17 [32] was also aligned for comparison. FNLG residues of *P. intermedia *17 were also underlined. The alignment analysis data from the UN *C. lari *isolates RM2100, 298, 300 and 84C-1, from the UPTC isolates NCTC12892, 12893, 12895, 12896, CF89-12, A1, A2, A3, 89049 and 92251, and from *C. jejuni *strains RM1221, 81-176, 260.94, CF93-6, HB93-13, 8425 and ss doylei 269.97 were omitted from the Figure, because of the occurrence of the identical sequences.

When, in retation to a single Fn-binding domain localized at four amino acid (FRLS; CadF amino acid positions 134-137 for *C. jejuni*) [28], amino acid sequence alignment analysis was carried out, the putative *cadF *(-like) ORFs from all 17 *C. lari *isolates examined showed amino acid residues of FALG (50% identity) within the amino acid positions 137-140 instead of the FRLS residues, as shown in Figure 4.

**Figure 5:**
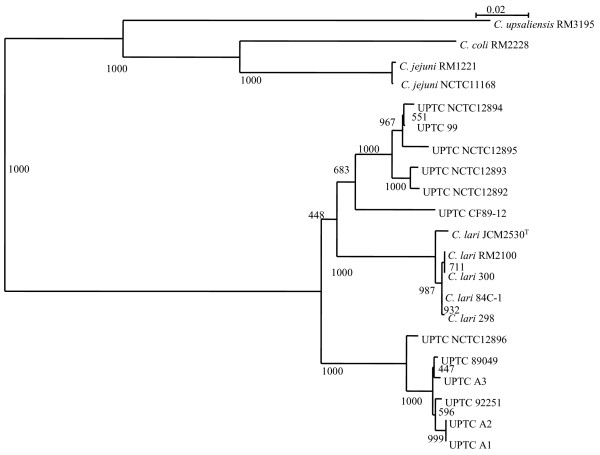
**A phylogenetic tree constructed based on nucleotide sequence information of full-length *cadF *(-like) gene from 17 *C. lari *isolates and other thermophilic campylobacters**. The tree was constructed by the NJ method [29]. values, 0.02, in the figure represent evolutionary distances. Boot-strap values of 1,000 are shown at the branch point. Out-group is *C. upsaliensis *RM3195.

A dendrogram showing phylogenetic relationships constructed by the NJ method [29] based on nucleotide sequence information of full-length *cadF *(-like) gene from 16 *C. lari *isolates and *C. lari *RM2100 and other thermophilic *Campylobacter *reference strains, the 17 *C. lari *isolates forming a major cluster separating from the other three thermophilic *Campylobacter *spp. (Figure 5). In addition, UN *C. lari *and UPTC organisms were not different and similar based on the nucleotide sequence data of the *cadF *(-like) gene, as shown in Figure 5.

## Discussion

This is the first demonstration of the structural analysis of the full-length gene encoding a CadF (-like) protein and its adjacent genetic loci within *C. lari*.

Regarding the NC region upstream of the *cadF *(-like) gene, this region is approximately 250 bp in length with all 16 *C. lari *isolates and *C. lari *RM2100 strain. However, the NC regions from the eight *C. jejuni *and a *C. coli *reference strains shown in Table 1 examined, are shorter than those and approximately 150 bp in length with unknown reason(s).

In 1995, Koebnik described a peptidoglycan-associating alpha-helical consensus motif in the C-terminal regions of 16 bacterial cell-surface proteins (NX_2 _LSX_2 _RAX_2 _VX_3_L) [30]. When we compared the corresponding amino acid sequences of the putative *cadF *(-like) ORF from the 17 *C. lari *and some *C. jejuni *isolates with this consensus motif, the motif was completely conserved amongst the *cadF *(-like) ORFs from the isolates (data not shown).

As shown in Table 2, the CMW of the putative *cadF *(-like) ORF was estimated to be 36,578 to 36,869 Da for the 16 *C. lari *isolates and *C. lari *RM2100 reference strain (data not shown). In addition, the value was also estimated to be approximately 36 kDa for the two *C. jejuni *reference strains (Table 2). These estimated CMW values are in agreement with the previous description of the immunodetection of the CadF protein from five *C. jejuni *and *C. coli *isolates [25].

When the nucleotide and deduced amino acid sequence alignment analyses were carried out for the putative *cadF *(-like) ORF, apparent size differences occurred amongst the four thermophilic *Campylobacter *species, as described above. Regarding the putative ORFs for *cadF *(-like) gene between *C. lari *and *C. jejuni *organisms, nine amino acid residues are shorter in *C. jejuni *strains than in *C. lari *isolates.

Recently, Krause-Gruszczynska *et al*. (2007) described that the CadF protein from *C. coli *strains was 13 amino acid larger than those from *C. jejuni *strains, based on the deduced amino acid sequence alignment analysis [31]. This is consistent with our present results (Table 2). They also indicated that *C. coli *strains carried a stretch of 13 amino acid in the middle region of the protein [31]. In addition, in the present study, the deduced CadF (-like) protein was shown to be 328 amino acid from all 17 *C. lari *isolates and were nine amino acid larger than CadF from two *C. jejuni *strains (319 amino acid) (Table 2). Then, we carried out deduced amino acid sequence alignment analysis to elucidate the differences in CadF (-like) protein between *C. lari *and *C. jejuni *organisms. As shown in Figure 3, the *C. coli *RM2228 strain carried a stretch of 12 amino acid (VVTPAPAPVVSQ) from amino acid positions 190 to 201 as well as a Q at amino acid position 180 (Figure 3). In relation to the nine larger amino acid for *C. lari *isolates than *C. jejuni *strains, interestingly, four amino acid sequences (THTD) from amino acid positions 80 to 83 and five [A(T for UPTC99) KQID] from 193 to 197 were identified, as shown in Figure 3.

Regarding the CadF in *Campylobacter*, the *cadF *virulence gene, encoding 37 kDa outer membrane protein that promotes the binding of the pathogens to intestinal epithelial cells, was identified and cloned [22,25]. In relation to identification of the binding domain within *C. jejuni *CadF, Konkel *et al*. (2005) recently described that a single Fn-binding domain was localized at four amino acids (CadF amino acid positions 134 -137), consisting of the residues, phenylalanine-arginine-leucine-serine (FRLS) [28]. However, when amino acid sequence alignment analysis was carried out, the putative *cadF *(-like) ORFs from all 17 *C. lari *isolates examined in the present study showed amino acid residues of FALG (50% identity) within the amino acid positions 137 - 140, instead of the FRLS residues (Figure 4). No FRLS residues were also detected within any other regions of the *cadF *(-like) ORF from all 17 *C. lari *isolates examined. Interestingly, FNLG residues within AdpB (Ad-adhesin in p-*Prevotella*, B-second identified adhesin) in *Prevotella intermedia *(a black-pigmented gram-negative anaerobe) [32] was 75% identical to the FALG from *C. lari *(Figure 4). Therefore, it may be important to clarify if the *CadF *(-like) protein from *C. lari *isolates can bind to fibronectin or not. An experiment is now in progress to resolve this.

In the present study, for the first time, we have described the cloning, sequencing and characterization of full-length Cla_0387 from the 16 *C. lari *isolates. The CMW values were estimated to be 23,689 - 23,875 Da for the 16 *C. lari *isolates and *C. lari *RM2100 strain and these values were also equivalent to those from two *C. jejuni *and a *C. coli *reference strains (Table 2). In addition, the *cadF *(-like) gene and the Cla_0387 gene may possibly be functional within *C. lari *isolates, based on the present northern blot hybridization and RT-PCR observations, as shown in Figure 2A and 2B.

Thus, the *cadF *(-like) gene and the Cla_0387 gene could be co-transcribed within *C. lari *organisms, consisting of an operon. Since the Cla_0387 showed a high deduced amino acid sequence similarity to the *Escherichia coli *haloacid dehalogenase-like phosphatase [33], these two may have an important biological relationship within the *C. lari *cells.

In the present study, the authors designed two novel primer pairs (f-/r-*cadF*1 and f-/r-*cadF*2) *in silico *for amplification of an approximate 2.3 kbp region, including the full-length *cadF *(-like) gene and its adjacent genetic loci, based on sequence information of *C. lari *RM2100, *C. jejuni *RM1221 and *C. coli *RM2228 strains, resulting in successful amplification, TA-cloning and sequencing of those from the 16 *C. lari *isolates isolated from differencet sources and in several countries. Therefore, the present novel PCR primer pairs would be likely of value for, *C. jejuni *and *C. coli *organisms, as well as for other *C. lari *isolates.

A dendrogram showing phylogenetic relationships was constructed by the NJ method [29], based on nucleotide sequence information of full-length *cadF *(-like) gene from 16 *C. lari *isolates and *C. lari *RM2100 and other thermophilic *Campylobacter *reference strains. As shown in Figure 5, the 17 *C. lari *isolates form a major cluster separating from the other three thermophilic *Campylobacter *spp. In addition, the 17 *C. lari *isolates form some minor clusters, respectively, based on nucleotide sequence information from *cadF *(-like) gene (Figure 5). Thus, nucleotide sequence information of full-length *cadF *(-like) gene can be regarded as reliable in the molecular discrimination of *C. lari *organisms from the other three thermophilic campylobacters. In addition, Figure 5 also indicated that NJ dendrogram of UN *C. lari *and UPTC organisms were not different and similar based on the nucleotide sequence data of the *cadF*-like gene.

## Conclusion

The combined sequences encoding a partial and putative *rpsI *open reading frame (ORF), non-coding (NC) region, a putative ORF for the *Campylobacter *adhesin to fibronectin-like gene, a putative Cla_0387 ORF, NC region and a partial and putative Cla_0388 ORF, were identified in 16 *Campylobacter lari *isolates, using two novel degenerate primer pairs. Transcription of the *cadF*-like gene in *C. lari *cells *in vivo *was also confirmed and the transcription initiation site was determined. The putative *cadF *(-like) ORFs from all *C. lari *isolates were nine amino acid larger than those from *C. jejuni*, and showed amino acid residues 137 -140 of FALG (50% identity), instead of the FRLS residues of the maximal fibronectin-binding activity site demonstrated within *C. jejuni *CadF.

## Methods

### *Campylobacter *isolates and culture conditions

*C. lari *isolates (n = 4 UN *C. lari*; n = 12 UPTC), which were isolated from different sources and in several countries of Asia, Europe and North America and used in the present study, are shown in Table 1.

These isolates were cultured on Mueller-Hinton broth medium at 37°C for 48 h in an aerobic jar on Blood Agar Base No. 2 (Oxoid, Hampshire, UK) containing 7% (v/v) defibrinated horse blood (Nippon Bio-Test, Tokyo, Japan) and *Campylobacter *selective medium (Virion, Zurich, Switzerland). An atmosphere of 5% (v/v) O_2 _and 10% (v/v) CO_2 _was produced by BBL Campypak Microaerophilic System Envelopes (Bacton Dickinson, NJ, USA).

### Genomic DNA preparation, primer design and PCR amplification

Genomic DNA was prepared using sodium dodecyl sulfate and proteinase K treatment, phenol-chloroform extraction and ethanol precipitation [34].

Two novel degenerate primer pairs (f-/r-*cadF*1 and f-/r-*cadF*2) were first designed *in silico *to generate two PCR products of approximately 1.4 and 1.2 kbp respectively, corresponding to the full-length *cadF*-like gene and its adjacent genetic loci, including full-length Cla_0387 (approximately 2.3 kbp) for the *C. lari *isolates, based on the sequence information of *C. lari *RM2100, *C. jejuni *RM1221 and *C. coli *RM2228 strains. A schematic representation of the *cadF *gene and its adjacent genetic loci for *C. lari *RM2100 (AAFK01000002) [26], including the locations of the two primer pairs and nucleotide sequences of the primers designed *in silico *in the present study, are shown in Figure 1.

PCR mixtures contained 50 ng of template DNA, 10 mM Tris-HCl pH 8.3, 50 mM KCl, 1.5 mM MgCl_2_, 400 μM of each dNTP, 0.6 μM of each primer, and a total of 1 unit of rTaq DNA polymerase (Takara Bio Inc., Shiga, Japan). PCR was performed in 50 μl reaction volumes, for 30 cycles of 94°C for 1.0 min, 50°C for the f-/r-*cadF*1 and 45°C for the f-/r-*cadF*2 for 0.5 min, and 72°C for 1.5 min, followed by a final extension at 72°C for 5.0 min.

### TA cloning, nucleotide sequencing and sequence analyses

Amplified PCR products were separated by 1.0% (w/v) agarose gel electrophoresis in 0.5× TBE at 100 V and detected by staining with ethidium bromide. PCR products amplified by the newly constructed two primer pairs were purified using a QIAquick PCR Purification Kit (QIAGEN, Tokyo, Japan) and inserted into the pGEM-T vector using the pGEM-T Easy Vector System (Promega Corp. Tokyo, Japan). Sequencing of the cloned *cadF *(-like) gene fragments was performed with a Hitachi DNA autosequencer (SQ5500EL; Hitachi Electronics Engineering Co. Tokyo, Japan), after dideoxy nucleotide sequencing using a Thermo Sequenase Pre-Mixed Cycle Sequencing Kit (Amersham Pharmacia Biotech, Tokyo, Japan). Sequence analysis of the PCR amplicons was carried out using the computer software GENETYX-MAC version 9 (GENETYX Co., Tokyo, Japan).

### Total cellular RNA purification, reverse transcription-PCR, northern blot hybridization and primer extension analysis

Total cellular RNA was extracted and purified from *C. lari *cells by using RNA protect Bacteria Reagent and RNeasy Mini Kit (QIAGEN). Reverse-transcription (RT)-PCR was carried out with a primer pair of f-*cadF*2 and r-*cadF*3 (Figure 1), by using the QIAGEN OneStep RT-PCR Kit (QIAGEN). This primer pair is expected to generate a RT-PCR product of the *cadF *(-like) structural gene segment of approximately 780 bp including the Cla_0387 region. Northern blot hybridization analysis was carried out according to the procedure described by Sambrook and Russell (2001) [34], using a PCR amplified *cadF *(-like) fragment as a probe. The fragment was amplified using a primer pair of f-/r-*cadF*4 (Figure 1). Random primer extension was performed in order to prepare the fragment probe using a DIG-High Prime (Roche Applied Science, Penzberg, Germany).

The transcription initiation site for the *cadF *(-like) gene was determined by the primer extension analysis with the purified total cellular RNA of *C. lari *JCM2530^T ^cells. The primer that was selected for this assay was 5'-CTAAATTTCCTTCTGGMGTTGT-3', which corresponds to the reverse complementary sequence of np 504 through 525. The transcription initiation site was determined by primer extension with the sizes of DNA fragments generated by sequencing reactions. In the present study, the np which the authors used, are for those of *C. lari *JCM2530^T^.

### Phylogenetic analysis

Nucleotide sequences of approximately 980 bp of the full-length *cadF *(-like) gene, from the isolates of *C. lari *and the *C. lari *RM2100 strain, were compared to each other and with the accessible sequence data from some other thermophilic campylobacters using CLUSTAL W software, respectively [35], which was incorporated in the DDBJ. Following this, a phylogenetic tree was constructed by the neighbor-joining (NJ) method [29].

## List of abbreviations used

CadF: *Campylobacter *adhesin to fibronectin; *C. lari*: *Campylobacter lari*; kbp: kilo base pairs; np: nucleotide position; ORF: open reading frame; RT: reverse transcription; UPTC: urease-positive thermophilic *Campylobacter*; UN: urease-negative.

## Authors' contributions

JH, TS, AT and IT were involved with cloning, sequencing and analysis of the rRNA gene sequences from *Campylobacter *strains. JEM and BCM participated in its design and coordination, and review of the manuscript. MM participated in design of the study, collected strains, drafted the manuscript and reviewed the manuscript. All authors read and approved the final version.

## References

[B1] Benjamin J Leaper S Owen RJ Skirrow MB Description of Campylobacter laridis, a new species comprising the nalidixic acid resistant thermophilic Campylobacter (NARTC) group Curr Microbiol 1983 8 231 238 10.1007/BF01579552

[B2] Blaser MJ Taylor DN Feldman RA Epidemiology of Campylobacter jejuni infections Epidemiol Rev 1983 5 157 176 6357819 10.1093/oxfordjournals.epirev.a036256

[B3] Stirling J Griffith M Blair I Cormican M Dooley JSG Goldsmith CE Glover SG Loughrey A Lowery CJ Matsuda M McClurg R McCorry K McDowell D McMahon A Millar BC Nagano Y Rao JR Rooney PJ Smyth M Snelling WJ Xu J Moore JE Prevalence of gastrointestinal bacterial pathogens in a population of zoo animals Zoo Public Health 2008 55 166 172 10.1111/j.1863-2378.2007.01099.x 18331520

[B4] Skirrow MB Benjamin J '1001' campylobacters: cultural characteristics of intestinal campylobacters from man and animals J Hyg (Camb) 1980 85 427 442 10.1017/s0022172400063506 PMC2134020 7462593

[B5] Martinot M Jaulhac B Moog R Martino SD Kehrli P Monteil H Piemont Y Campylobacter lari bacteremia Clin Microbiol Infect 2001 7 96 97 10.1046/j.1469-0691.2001.00212.x 11298152

[B6] Nachamkin I Stowell C Skalina D Jones AM Hoop RM Smibert RM Campylobacter laridis causing bacteremia in an immunosuppressed patient Ann Int Med 1984 101 55 57 6375505 10.7326/0003-4819-101-1-55

[B7] Simor AE Wilcox L Enteritis associated with Campylobacter laridis J Clin Microbiol 1987 25 10 12 3793864 10.1128/jcm.25.1.10-12.1987 PMC265800

[B8] Tauxe RV Patton CM Edmonds P Brenner DJ Blake PA Illness associated with Campylobacter laridis, a newly recognized Campylobacter species J Clin Microbiol 1985 21 222 225 3972989 10.1128/jcm.21.2.222-225.1985 PMC271617

[B9] Werno AM Klena JD Shaw GM Murdoch DR Fatal case of Campylobacter lari prosthetic joint infection and bacteremia in an immunocompetent patient J Clin Microbiol 2002 40 1053 1055 10.1128/JCM.40.3.1053-1055.2002 11880437 PMC120280

[B10] Bolton FJ Holt A Hutchinson DN Urease-positive thermophilic campylobacters Lancet 1985 I 1217 1218 10.1016/S0140-6736(85)92898-3 2860418

[B11] Mégraud F Chevrie D Desplaces N Sedallian A Guesdon JL Urease-positive thermophilic *Campylobacter *(*Campylobacter laridis *variant) isolated from an appendix and from human feces J Clin Microbiol 1988 26 1050 1051 3384898 10.1128/jcm.26.5.1050-1051.1988 PMC266518

[B12] Owen RJ Costas M Sloss L Bolton FJ Numerical analysis of electrophoretic protein patterns of Campylobacter laridis and allied thermophilic campylobacters from the natural environment J Appl Bacteriol 1988 65 69 78 3209518 10.1111/j.1365-2672.1988.tb04319.x

[B13] Bezian MC Ribou G Barberis-Giletti C Megraud F Isolation of a urease positive thermophilic variant of Campylobacter lari from a patient with urinary tract infection Eur J Clin Microbiol Infect Dis 1990 9 895 897 10.1007/BF01967506 2073901

[B14] Kaneko A Matsuda M Miyajima M Moore JE Murphy PG Urease-positive thermophilic strains of Campylobacter isolated from seagulls (Larus spp.) Lett Appl Microbiol 1999 29 7 9 10.1046/j.1365-2672.1999.00565.x 10432626

[B15] Matsuda M Kaneko A Stanley T Miller BC Miyajima M Murphy PG Moore JE Characterization of urease-positive thermophilic Campylobacter subspecies by multilocus enzyme electrophoresis typing Appl Environ Microbiol 2003 69 3308 3310 10.1128/AEM.69.6.3308-3310.2003 12788730 PMC161486

[B16] Wilson IG Moore JE Presence of Salmonella spp. and Campylobacter spp. in shelfish Epidemiol Infect 1996 116 147 153 10.1017/S0950268800052377 8620905 PMC2271625

[B17] Endtz HP Vliegenthart JS Vandamme P Waverink HW Braak NP Van den Verbrug HA Belkum AV Genotypic diversity of Campylobacter lari isolated from mussels and oysters in The Netherlands Int J Food Microbiol 1997 34 79 88 10.1016/S0168-1605(96)01174-9 9029257

[B18] Matsuda M Kaneko A Fukuyama M Itoh T Shingaki M Inoue M Moore JE Murphy PG Ishida Y First finding of urease-positive thermophilic strains of Campylobacter in river water in the Far East, namely, in Japan, and their phenotypic and genotypic characterization J Appl Bacteriol 1996 81 608 612

[B19] Matsuda M Shibuya T Itoh Y Takiguchi M Furuhata K Moore JE Murayama O Fukuyama M First isolation of urease-positive thermophilic Campylobacter (UPTC) from crows (Coruvs levaillantii) in Japan Int J Hyg Environ Health 2002 205 321 324 10.1078/1438-4639-00157 12068751

[B20] Matsuda M Moore JE Urease-positive thermophilic Campylobacter species Appl Environ Microbiol 2004 70 4415 4418 10.1128/AEM.70.8.4415-4418.2004 15294767 PMC492433

[B21] Fröman G Switalski LM Faris A Wadstom T Hook M Binding of Escherichia coli to fibronectin J Biol Chem 1984 259 14899 14905 6238965

[B22] Konkel ME Garvis SG Tipton SL Anderson DE Jr Cieplak W Jr Identification and molecular cloning of a gene encoding a fibronectin-binding protein (CadF) from Campylobacter jejuni Mol Microbiol 1997 24 953 963 10.1046/j.1365-2958.1997.4031771.x 9220003

[B23] Myhre EB Kuusela P Binding of human fibronectin to group A, C, and G streptococci Infect Immun 1983 40 29 34 6299959 10.1128/iai.40.1.29-34.1983 PMC264813

[B24] van Putten JP Duensing TD Cole RL Entry of OpaA+ gonococci into HEp-2 cells requires concerted action of glycosaminoglycans, fibronectin and integrin receptors Mol Microbiol 1998 29 369 379 10.1046/j.1365-2958.1998.00951.x 9701828

[B25] Konkel ME Gray SA Kim BJ Garvis SG Yoon J Identification of the enteropathogens Campylobacter jejuni and Campylobacter coli based on the *cadF *virulence gene and its product J Clin Microbiol 1999 37 510 517 9986804 10.1128/jcm.37.3.510-517.1999 PMC84446

[B26] Fouts DE Mongodin EF Mandrell RE Miller WG Rasko DA Ravel J Brinkac LM Deboy RT Major structural differences and novel potential virulence mechanisms from the genomes of multiple Campylobacter species PLoS Biol 2005 3 e15 10.1371/journal.pbio.0030015 15660156 PMC539331

[B27] Benjamin L Genes VII 2000 Oxford; Oxford University Press

[B28] Konkel ME Christensen JE Keech AM Monteville MR Klena JD Garvis SG Identification of a fibronectin-binding domain within the Campylobacter jejuni CadF protein Mol Microbiol 2005 57 1022 1035 10.1111/j.1365-2958.2005.04744.x 16091041

[B29] Saitou N Nei M The neighbor-joining method: a new method for reconstructing phylogenetic tree Mol Biol Evol 1987 4 406 425 3447015 10.1093/oxfordjournals.molbev.a040454

[B30] Koebnik R Proposal for a peptidoglycan-associating alpha-helical motif in the C-terminal regions of some bacterial cell-surface proteins Mol Microbiol 1995 16 1269 1270 10.1111/j.1365-2958.1995.tb02348.x 8577259

[B31] Krause-Gruszczynska M van Alphen LB Oyarzabal OA Alter L Hanel I schliephake A Konig W van Putten JM Konkel ME Expression patterns and role of the CadF protein in Campylobacter jejuni and Campylobacter coli FEMS Microbiol Lett 2007 274 9 16 10.1111/j.1574-6968.2007.00802.x 17573935

[B32] Yu F Lyer D Anaya C Lewis JP Identification and characterization of a cell surface protein of Prevotella intermedia 17 with broad spectrum binding activity for extra cellular matrix proteins Proteomics 2006 6 6023 6032 10.1002/pmic.200600177 17051640

[B33] Kuznetsova E Proudfoot M Gonzalez CF Brown G Omelchenko MV Borozan I Carmel L Wolf YI Mori H Savchenko AV Arrowsmith CH Koonin EV Edwards AM Yakunin AF Genome-wide analysis of substrate specificities of the Escherichia coli haloacid dehalogenase-like phosphatase family J Biol Chem 2006 47 36149 36161 10.1074/jbc.M605449200 16990279

[B34] Sambrook J Russell DW Molecular Cloning; a laboratory manual 2001 3 Cold Spring Harbor Laboratory Press., Cold Spring harbor, N. Y

[B35] Thompson JD Higgins DG Gibson TJ CLUSTAL W: improving the sensitivity of progressive multiple sequence alignment through sequence weighting, position-specific gap penalties and weight matrix choice Nucleic acids Res 1994 22 4673 4680 10.1093/nar/22.22.4673 7984417 PMC308517

